# Genome-Wide Identification of the bHLH Gene Family in *Callerya speciosa* Reveals Its Potential Role in the Regulation of Isoflavonoid Biosynthesis

**DOI:** 10.3390/ijms252211900

**Published:** 2024-11-06

**Authors:** Liuping Chen, Xiaoming Tan, Ruhong Ming, Ding Huang, Yong Tan, Liangbo Li, Rongshao Huang, Shaochang Yao

**Affiliations:** 1 College of Pharmacy, Guangxi University of Chinese Medicine, Nanning 530200, China; chenliuping2022@stu.gxtcmu.edu.cn (L.C.); tanxm@gxtcmu.edu.cn (X.T.); huangd@gxtcmu.edu.cn (D.H.);; 2 Guangxi Key Laboratory of Zhuang and Yao Ethnic Medicine, Guangxi University of Chinese Medicine, Nanning 530200, China

**Keywords:** *C. speciosa*, genome-wide identification, bHLH transcription factor, isoflavonoid biosynthesis

## Abstract

*Callerya speciosa* (Champ. ex Benth.) Schot is a significant leguminous plant valued for its edible tuberous roots, which are a plentiful source of isoflavonoids. Basic helix–loop–helix (bHLH) transcription factors (TFs) have been reported to regulate secondary metabolism in plants, especially flavonoid biosynthesis. However, the *bHLH* genes in *C. speciosa* have not yet been reported, and their regulatory role in isoflavonoid biosynthesis remains unexplored. Here, 146 *CsbHLH* genes were identified in the *C. speciosa* genome, classifying them into 23 subfamilies based on the gene structures and phylogenetic relationships. All the CsbHLH proteins contained both motifs 1 and 2, whereas motif 8 was only distributed in subgroup III (d + e). Collinearity analysis demonstrated that fragmental replications are the primary driver of CsbHLH evolution, with the majority of duplicated *CsbHLH* gene pairs experiencing selective pressure. Nine candidate *CsbHLH* genes were found to play a potential role in regulating isoflavonoid biosynthesis through a combination of gene-to-metabolite correlation analysis and weighted gene co-expression network analysis (WGCNA). Additionally, the *cis*-regulatory elements and response to MeJA of these nine genes were characterized and confirmed through quantitative real-time PCR (qRT-PCR) analysis. Among them, three CsbHLHs (CsbHLH9, CsbHLH89, and CsbHLH95) were selected for further investigation. Yeast two-hybrid (Y2H), dual-luciferase (LUC) assays, bimolecular fluorescence complementation (BiFC) assays, and transient transformation demonstrated that CsbHLH9 acted as a transcriptional activator through its interaction with CsMYB36 and binding to the promoters of isoflavonoid biosynthesis genes in a MeJA-induced manner, such as *CsIFR2*, *CsI3′H2*, and *CsCHS4*, to promote isoflavonoid (calycosin, calycosin-7-o-glucoside, and formononetin) accumulation. Our results establish a basis for the functional analysis of *bHLH* genes and investigations into the molecular mechanisms underlying isoflavonoid biosynthesis in *C. speciosa*.

## 1. Introduction

Along with the rapid development of high-speed sequencing technologies, there is a growing recognition of the vital functions played by expanding transcription factor (TF) families in higher plants to function in regulating their growth processes and metabolite accumulation, such as the basic helix–loop–helix (bHLH), WD40, myeloblastosis (MYB), WRKY, and basic leucine zipper (bZIP) families [1]. The bHLH TF family is acknowledged as a prominent and essential group in eukaryotic organisms, distinguished by the conserved bHLH domain. Increasing bHLH TFs have been recognized with the help of genome databases, with 162, 167, and 111 bHLH genes identified in *Arabidopsis* (*Arabidopsis thaliana*) [2,3], rice [4], and *Gynostemma pentaphyllum* [5], respectively. The bHLH domain comprises around 60 amino acids, organizing two conserved motifs: a basic region and an HLH region. The basic region located in the N-terminus typically includes a segment of 10–15 amino acids and harbors DNA *cis*-acting elements such as the E-box (CANNTG) and G-box (CACGTG) motifs to support interactions with their target genes [6]. The HLH region, spanning approximately 40 amino acids in the C-terminus, comprises two amphipathic α-helices connected by a loop and enriched with hydrophobic residues. This domain is anticipated to function as the dimerization domain, modulating the transcription of target genes by facilitating the assembly of homo- or heterodimers [7,8].

Eukaryotic bHLH proteins are commonly categorized into distinct subfamilies or subgroups by researchers through the consideration of factors such as phylogenetic relatedness, DNA-binding specificity, and the conservation of domains. A total of 638 bHLH genes sourced from *Arabidopsis*, *Populus trichocarpa*, *Oryza sativa*, *Physcomitrella patens*, and five algae species were allocated into 32 subfamilies. In brief, subfamilies 9 and 27 play a crucial role in the growth and development of land plants, while subfamilies 7, 18, 19, and 20 are specific to flowering plants. Subfamily 5 members are commonly associated with the regulation of flavonoid/anthocyanin metabolism, epidermal cell fate determination, and trichome initiation [9]. At least 162 bHLH members are identified from the genome sequences, which are categorized into 12 distinctive subgroups (I–XII) in *Arabidopsis* [2,3]. Plant bHLH proteins form a monophyletic group and are classified into 26 subfamilies, with many distinguished by the presence of conserved short amino acid motifs [8]. The 152 bHLH genes in tomato [10] and 95 bHLH genes in *Ficus carica* [11] are divided into second-level subfamilies by the same method. Additionally, analysis of the promoter regions of Myc-like regulatory factors in wheat indicates their categorization into two groups: potentially homoeologous sets TaMyc-1 (including TaMyc-A1/TaMyc1, TaMyc-B1, TaMyc-D1) and TaMyc-2 (comprising TaMyc-A2 and TaMyc-D2). It is suggested that TaMyc-B1 may act as a co-regulator of the TaC1-A1 gene (encoding an R2R3-Myb factor) within the MBW regulatory complex, which triggers the activation of anthocyanin synthesis [12].

An in-depth investigation into plant bHLH transcription factors has unveiled their crucial involvement in a wide array of vital physiological functions, encompassing the regulatory control of pivotal developmental pathways, such as carbohydrate metabolism during pollen development [13], stomatal differentiation [14], fiber development [15], and carpel and fruit development [16]. In addition, certain *bHLH* genes participate in the defense against diverse biotic and abiotic stresses through their interaction with the promoters of target genes, including those responsive to salt stress [17], iron deficiency [18], drought and salinity [19], and low temperature [20]. Furthermore, previous studies have demonstrated that distinct members of the bHLH family are involved in modulating the flavonoid biosynthetic pathway in plants through the assembly of the R2R3-MYB, bHLH, and WD40 repeat (MBW) protein complex [21]. For instance, PPLS1, a bHLH transcription factor, interacts with SiMYB85 to control anthocyanin production in *Setaria italica* [22]. Strawberry FaEGL3 and FaLWD1/FaLWD1-like proteins interact with R2R3-FaMYB5 to assemble an MYB-bHLH-WD40 complex (MBW) which augments regulatory efficacy [23].

*Callerya speciosa* (Champ. ex Benth.) Schot, also known as ‘niudali’ in Chinese, has been widely used not only as a traditional food but also as a traditional Chinese medicinal herb in tropical and subtropical countries. In South China, the cultivating areas of *C. speciosa* reached approximately 15,000 hm^2^ in 2024 and were rapidly increasing along with the booming of the health industry. The tuberous roots are rich in bioactive compounds such as flavonoids, coumarins, alkaloids, and terpenes [24]. These components contribute significantly towards hepatoprotection, anti-oxidation, anti-inflammation, anti-tumor, and immunity-enhancing activities, as well as regulating glycolipid metabolism [24,25,26,27]. Isoflavonoids, notably formononetin and maackiain, are employed as indicative compounds to assess the quality of *C. speciosa* [28]. The quantification of isoflavonoid content is a multifaceted trait influenced by numerous genes that give rise to intricate regulatory networks [29]. Given that the isoflavonoid biosynthesis pathway has been extensively characterized, many researchers attempted to modify specific structural genes in the isoflavonoid biosynthetic pathway by genetic engineering in order to improve the content of isoflavonoids, but the results have shown no significant effects [30]. The identification and application of TFs specific to the (iso)flavonoid biosynthetic pathway may effectively resolve this problem. In *Epimedium sagittatum*, EsMYB9 interacts with EsTT8 or the AtTT8 bHLH regulator to regulate the flavonoid biosynthetic pathway [31]. The expression patterns of *PlMYB1*, *PlHLH3-4*, and *PlWD40-1* genes exhibited a strong correlation with the accumulation patterns of isoflavonoids in *Pueraria lobate* [32]. Our previous study has demonstrated that CsMYB36 was likely the key TF that regulated isoflavonoid biosynthesis in *C. speciosa* [28]. Nevertheless, the bHLH TFs associated with the biosynthesis of isoflavonoids and whether CsbHLHs interacted with CsMYB36 in *C. speciosa* remain unidentified.

In this study, a total of 146 *CsbHLH* genes in *C. speciosa* were recruited using a publicly available whole-genome database. Protein properties, phylogeny, gene structure and localization, conserved domains, motifs composition, *cis*-acting elements analysis, expression patterns, and protein interactions were analyzed using the bioinformatics method. The expression patterns of nine candidate *bHLH* genes under methyl jasmonate (MeJA) treatment were also discussed. Yeast two-hybrid (Y2H), dual-luciferase assays (LUC), bimolecular fluorescence complementation (BiFC), and transient transformation further demonstrated that, as the regulator of isoflavonoid biosynthesis, CsbHLH9 could bind to and activate CsMYB36. Our results enhance the comprehension of the bHLH gene family in *C. speciosa* and establish a basis for delving deeper into the regulatory role of *CsbHLHs* in isoflavonoid biosynthesis.

## 2. Results

### 2.1. Genome-Wide Identification of CsbHLH Genes and Their Phylogenetic Analysis

Following the elimination of redundant sequences, a thorough comparative analysis revealed the presence of 146 proteins encoding *bHLH* genes from the published genome of *C. speciosa*, named CsbHLH1–CsbHLH146. The proteins varied in size from 92 to 790 amino acids, with an average length of 357 amino acids. The molecular weights of CsbHLH proteins ranged from 10.21 (CsbHLH91) to 84.86 (CsbHLH68) kDa, while the theoretical pI values varied from 4.62 (CsbHLH109) to 10.65 (CsbHLH83), with the majority (41.78%) falling below seven. The Instability Index (II) ranged from 34.33 to 83.37, with only ten CsbHLH proteins classified as stable (II < 40). The change trends of the aliphatic index (AI) were from 55.60 to 103.99, with an average of 75.31. The grand average of hydropathicity (GRAVY) of the CsbHLH proteins ranged from −0.96 (CsbHLH6) to 0.01 (CsbHLH144), indicating that all CsbHLHs except for CsbHLH144 are hydrophilic. Subcellular localization prediction results revealed that the majority (87.67%) of CsbHLH proteins were predicted to localize in the nucleus, while cytoplasm and chloroplast were also predicted for a few of them, with percentages of 6.85% and 5.48%, respectively (Supplementary Table S2).

**Figure 1:**
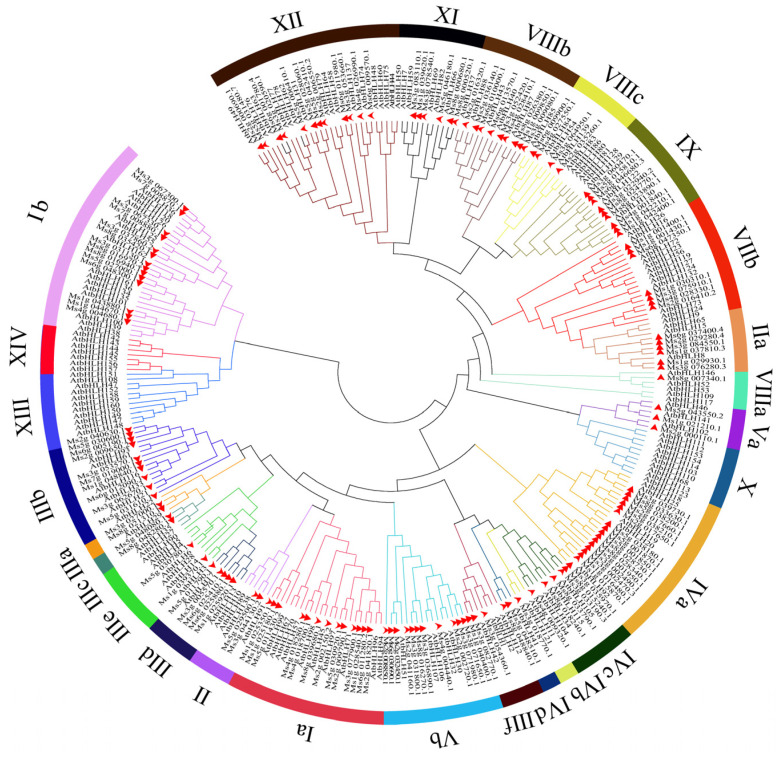
Phylogenetic tree and classification of bHLH proteins between *C. speciosa* and *Arabidopsis thaliana*. The unrooted evolutionary tree was constructed using the maximum likelihood (ML) method. The 146 CsbHLH proteins are categorized into 23 subfamilies under the classification of the 161 AtbHLH proteins belonging to 26 subfamilies, represented by different colored subfamilies within the phylogenetic tree. Different subfamilies are also marked with different background colors. The alphanumeric characters outside the main circle denote the name of each subfamily.

To elucidate the phylogenetic relationships of CsbHLH proteins, an unrooted maximum likelihood (ML) phylogenetic tree was constructed based on the taxonomy established for *A. thaliana* (Figure 1). The CsbHLHs were present in 23 of 26 highly conserved subgroups of *A. thaliana*; they were absent in subgroups X, XIV, and XIII. Subgroup IVa exhibited the highest abundance with 17 members, whereas subgroups IVb and VIIIa were the least populated, each comprising only one member. *C. speciosa* and *A. thaliana* shared an equal number of members in 13 subgroups, including IIIa, IIIc, IIIf, IVc, IVd, Va, Vb, VIIa, Ia, Ib, XII, VIIIc, and XI. The protein counts within the individual subgroups notably varied between *C. speciosa* and *A. thaliana*. For instance, the number of five subgroups (IIIb, IVa, IX, Vb, and VIIIb) in *C. speciosa* was obviously more than that of *A. thaliana* bHLH proteins. Contrastingly, CsbHLH had fewer members than *A. thaliana* in subgroups VIIb, X, XIV, and XIII. The results suggested that there were notable interspecific divergences observed within the *bHLH* gene family across the two species.

### 2.2. Conserved Domains, Motif Composition, and Gene Structural Characteristics of the CsbHLH Proteins

**Figure 2:**
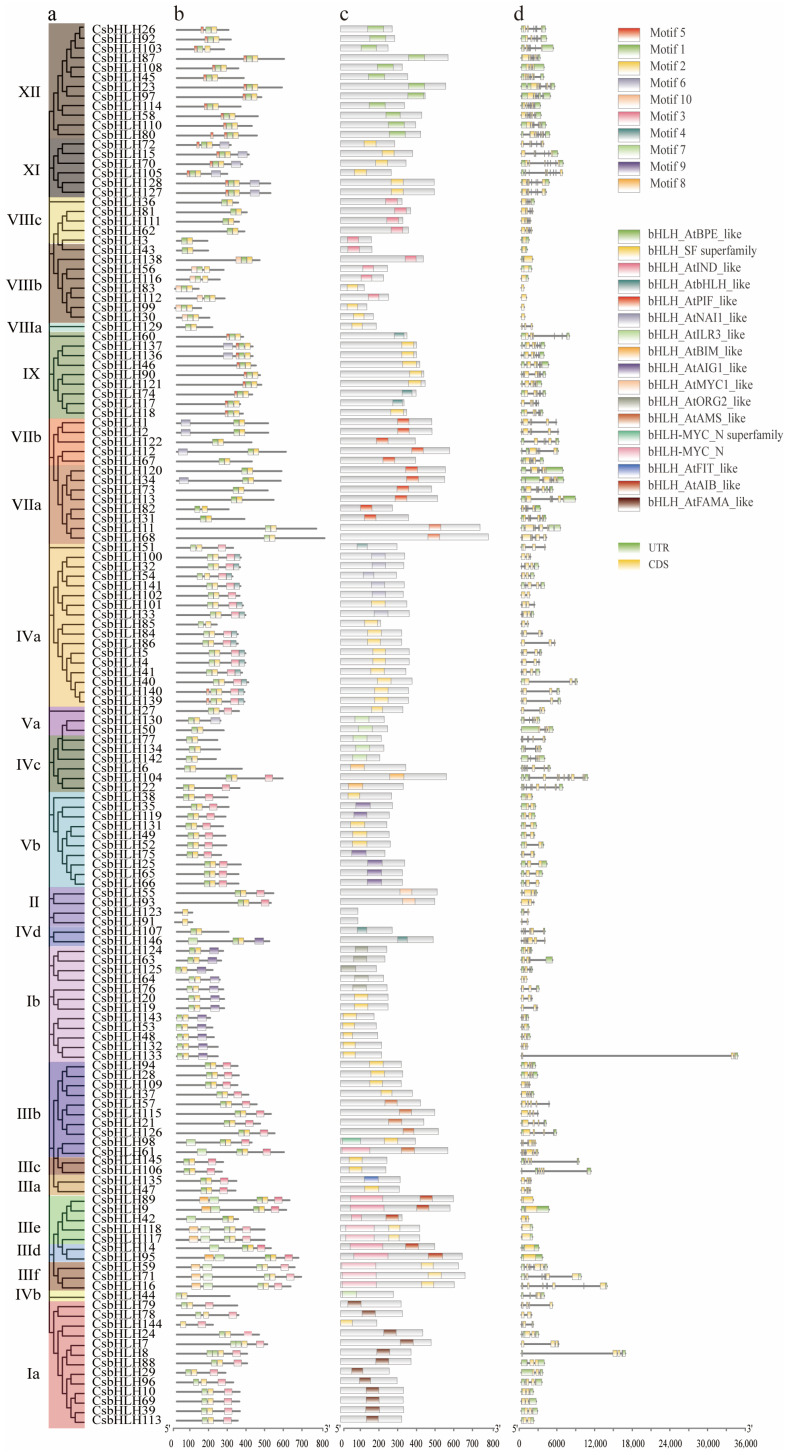
Analysis of phylogenetic relationships (**a**) motif composition (**b**) conserved domains (**c**) and gene structural characteristics (**d**) of CsbHLH proteins based on the phylogenetic relationships.

To verify the phylogenetic analysis of the *CsbHLH* gene family (Figure 2a), ten conserved motifs were identified using the online MEME tool (Supplementary Table S3). While there was notable variation in the length of CsbHLH proteins among different subfamilies, members within the same subgroups typically exhibited comparable patterns of motif composition and arrangement. Furthermore, certain subgroup-specific motifs were identified, potentially playing crucial roles in their distinct functions. For instance, all CsbHLH protein sequences were found to contain both motifs 1 and 2. All members of subgroups IVa (except CsbHLH85), Vb, III (a + b + c + d + f), and Ia were characterized by the presence of motif 3 (pink), while all members of subgroups XI, IX, and XII contained motif 5 (roes). Nonetheless, certain motifs were exclusive to specific subgroups. For example, motif 9 (light purple) was uniquely present in subgroup Ib, while motif 7 (light green) was identified in subgroup III, whereas motif 4 (light blue) was found in subgroup IVa. Motif 8 (orange) was only distributed in subgroup III (d + e), suggesting its potential significance in the biosynthesis of isoflavonoids (Figure 2b).

To elucidate the properties of the CsbHLH proteins in *C. speciosa*, the conserved domains among the members were analyzed using online software. All the members contained the bHLH domain, in which most of them contained the homologous domains with *A. thaliana*. Notably, members of subfamilies III (b + d + e + f) contained the MYC domain, whereas III (a + c) did not possess it (Figure 2c).

A comparative analysis of the exon-intron structure, including the number and positioning of exons and introns, of CsbHLH proteins was conducted by evaluating the respective genomic sequences (Figure 2d). Different numbers of exons were found within the 146 CsbHLH proteins, varying from 1 to 13. Eleven genes did not contain intron, and more than 80.82% of genes contained two or more introns.

### 2.3. Chromosomal Localization, Gene Duplication, and Synteny Analysis of the CsbHLH Proteins

**Figure 3:**
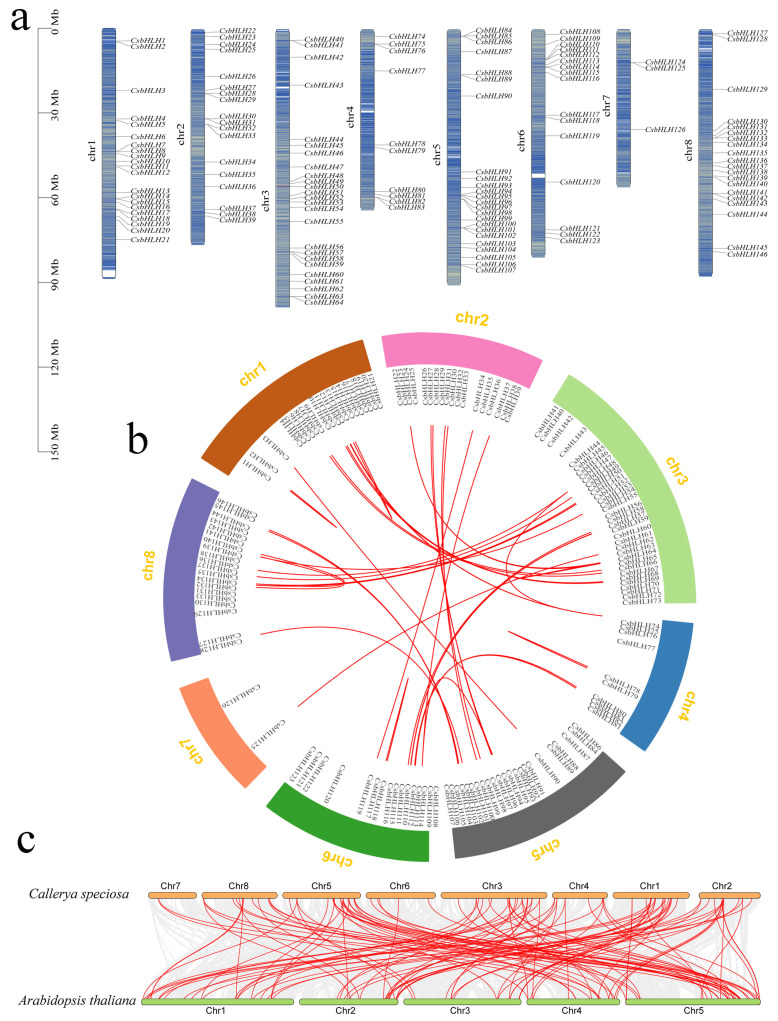
Chromosomal locations and collinearity analysis of *CsbHLH* genes (**a**) The *CsbHLH* genes are mapped on chromosomes; scale bar on the left represents the chromosome length (Mb) (**b**) The intra-and inter-species collinearity analysis. The collinear genes that have been identified are connected by red lines (**c**) Inter-species collinear analysis of *bHLH* genes between *C. speciosa* and *A. thaliana*. The highlight lines represent the syntenic *bHLH* gene pairs.

The chromosomal localization of *CsbHLH* genes was revealed, and the results showed that the 146 *CsbHLH* genes displayed an uneven distribution across the chromosomes, with chromosome 3 containing the highest number of genes (33, *CsbHLH40*–*CsbHLH73*) and chromosome 7 harboring the lowest number of genes (3, *CsbHLH124*–*CsbHLH126*) (Figure 3a). There was no observed association between the length of the chromosome and the quantity of *CsbHLH* genes present. To investigate the occurrence of gene duplication events in the evolutionary history of the *CsbHLH* gene family, an intraspecific collinearity analysis was conducted (Figure 3b). Tandem and fragment replication were significant processes in the expansion of the *CsbHLH* gene family, as indicated by Ka/Ks ratios < 1 for all homologous *CsbHLH* gene pairs, suggesting that these *CsbHLH* genes predominantly experienced purifying selection. It is worth noting that only three tandem replication clusters and fifty fragmental replication segments were identified from the *CsbHLH* gene family (Supplementary Table S4), indicating that fragmental replication might play more important roles in expanding the *CsbHLH* gene family.

A collinearity analysis utilizing MCScanX was carried out to identify synteny among *bHLH* genes in *C. speciosa* and *A. thaliana* (Figure 3c and Supplementary Table S5). It was observed that 117 paired collinearity relationships between 75 *CsbHLH* genes in *C. speciosa* and 75 *AtbHLH* genes in *A. thaliana* were found, indicating the *bHLH* gene family in *C. speciosa* exhibits a closely related homologous evolutionary relationship with *Arabidopsis*. Additionally, the orthologous genes in *C. speciosa* were mainly distributed in chromosomes 1, 3, and 5, suggesting that these chromosomes were relatively conserved in the revolution of *C. speciosa*.

### 2.4. Expression Pattern of CsbHLH Genes in Different Tissues

**Figure 4:**
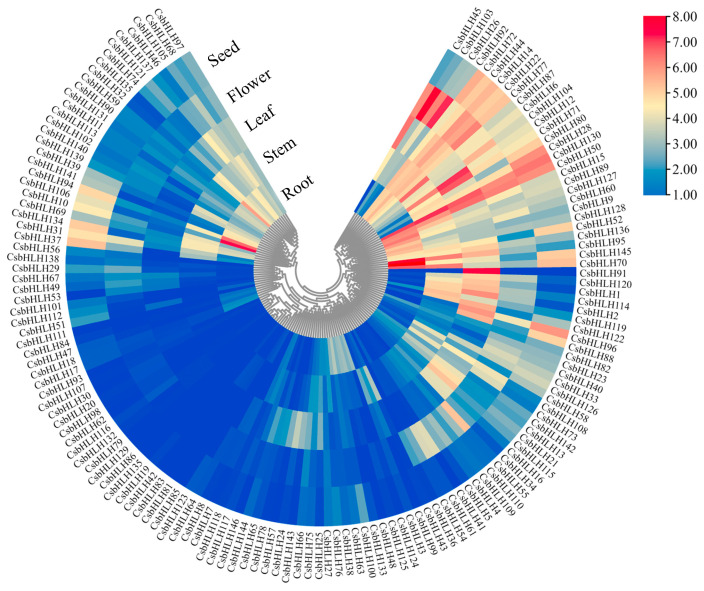
Expression profiles of *CsbHLH* genes in five different tissues. The log10-transformed expression values of FPKMs were calculated in hot map. Change in color from blue to red represents low to high expression level.

We harvested the root, stem, leaf, flower, and seed and then employed RNA-seq technology to analyze the expression of *CsbHLH* genes in these five tissues. Among the 146 *CsbHLH* genes, 71 exhibited the FPKM > 10 in one or more samples across the five tissues studied (Figure 4). Ten *CsbHLH* genes from eight subgroups identified FPKM values above 100, with subgroup III occupying four genes (*CsbHLH71*, *CsbHLH95*, *CsbHLH106*, and *CsbHLH145*). Three of these genes were obviously up-regulated in the root tissue, except that *CsbHLH71* had a high expression level in the leaf. Additionally, distinct expression profiles may be observed among members of a shared subfamily: in subfamily III (c + d + e), for example, *CsbHLH9*, *CsbHLH89*, *CsbHLH95*, *CsbHLH106*, and *CsbHLH145* were specifically high-expressed in the root, whereas *CsbHLH14* and *CsbHLH118* exhibited high levels of expression in the flower and stem, respectively. Four members of subfamily XI (*CsbHLH15*, *CsbHLH70*, *CsbHLH127*, and *CsbHLH128*) were highly expressed in the root, but *CsbHLH72* and *CsbHLH105* had a high expression level in other tissues. Six of the ten IVa subgroup members, especially CsbHLH32, were highly expressed in the roots.

The biosynthesis of isoflavonoids occurs exclusively in the roots of *C. speciosa*, playing a crucial role in improving its overall quality [28]. To elucidate the structural genes and TFs responsible for governing the biosynthesis of root-specific isoflavonoids, a weighted gene co-expression network analysis (WGCNA) was conducted using transcriptomic data derived from five distinct tissues (Figure S2). In total, ten modules displaying similar expression patterns were identified. Among them, the Meblack modules showed a positive correlation with the root (r > 0.9) and were regarded as a potential isoflavonoid-related module. In this module, a total of 24 bHLH TFs were identified from the Meblack modules (Supplementary Table S6), in which 9 *CsbHLH* genes (*CsbHLH9*, *CsbHLH15*, *CsbHLH32*, *CsbHLH89*, *CsbHLH95*, *CsbHLH106*, *CsbHLH127*, *CsbHLH128*, and *CsbHLH145*) exhibited particularly high expression levels in the root tissues and were regarded as the key candidate genes for further study. Additionally, 15 MYB and 8 MYB-related TFs were also identified from the Meblack modules, which showed a high positive significance with CsbHLHs, suggesting that they might function with CsbHLHs by forming a protein complex (Supplementary Table S6).

### 2.5. The Isoflavonoid-Related CsbHLH Genes and Their Cis-Element Analysis

**Figure 5:**
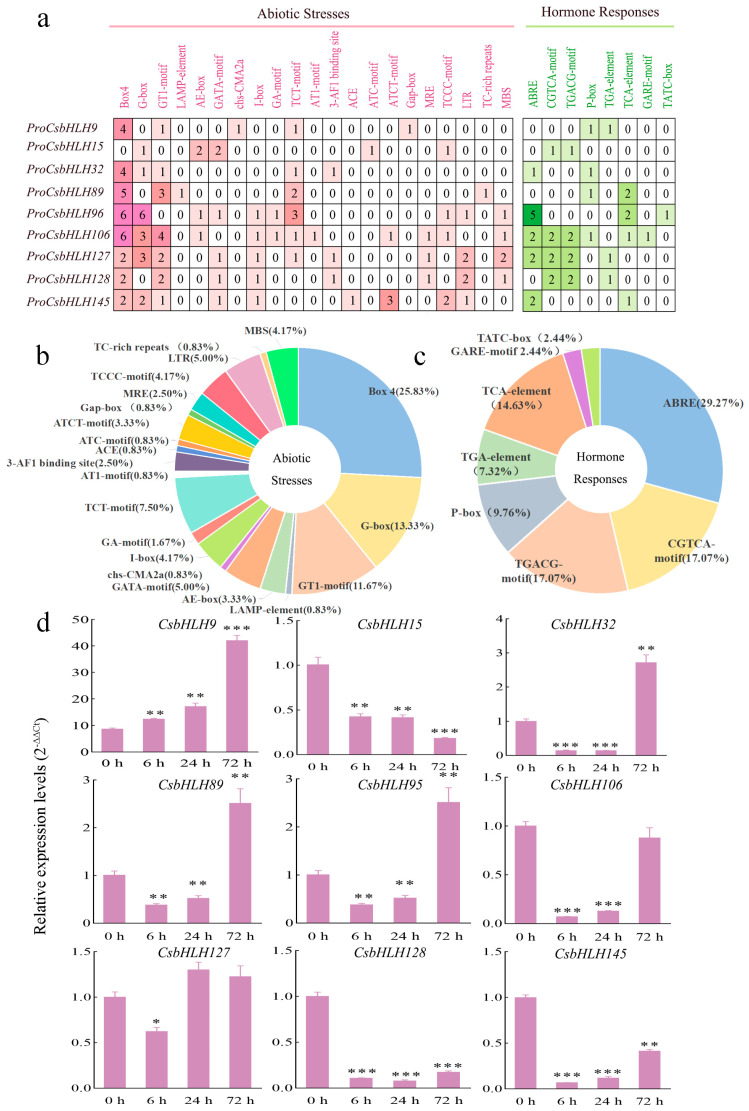
Promoter *cis*-elements analysis of the nine isoflavonoid-related CsbHLH genes. (**a**) The identified *cis*-acting elements were categorized into two functional groups, which are represented by red and green color shades. The numbers indicate the quantity of corresponding *cis*-acting elements in each CsbHLH gene promoter region, showing the distribution of *cis*-acting elements with various categories within abiotic stresses (**b**) and hormone response (**c**) with specific functions. (**d**) The relative expression profiles of nine CsbHLH genes were analyzed using qRT-PCR in response to MeJA treatments. Values represent the mean ± SD. Significant variations were determined by the Student’s *t*-test (* *p* < 0.05, ** *p* < 0.01, *** *p* < 0.001).

To explore the potential *cis*-regulatory elements associated with isoflavonoid biosynthesis, 2000 bp sequences upstream of the promoter region were retrieved for the nine *CsbHLH* genes and analyzed using the PlantCARE online tool for *cis*-element prediction. Two groups were categorized according to the *cis*-acting elements’ functions: abiotic stresses (light, low temperature, defense, and stress) and hormone responses (abscisic acid, auxin, gibberellin, salicylic acid, and methyl jasmonate) (Figure 5a). In the abiotic stresses group, a total of 21 subgroups were distinguished, with box4 emerging as the predominant *cis*-element at 25.83%, followed by G-box at 13.33%. The CGTCA-motif and TGACG-motif were identified as the predominant MeJA-responsive elements, totally occupying 34.14%, whereas ABRE (ABA-responsive element) accounted for 29.27% in the hormone responses group (Figure 5b,c). Four genes (*CsbHLH15*, *CsbHLH106*, *CsbHLH127*, and *CsbHLH128*) contained MeJA-responsive elements, suggesting that they might be influenced by MeJA. To test this proposition, we quantified the transcript levels of the nine *CsbHLHs* in MeJA-treated roots of *C. speciosa* using quantitative real-time PCR (qRT-PCR) analysis. As shown in Figure 5d, five *CsbHLHs* (*CsbHLH9*, *CsbHLH32*, *CsbHLH89*, *CsbHLH95*, and *CsbHLH127*) demonstrated significant up-regulation in response to MeJA in comparison to the control group, while others were inhibited by MeJA. Based on our previous results [33] and their response to MeJA, we hypothesize that these MeJA-triggered *CsbHLHs* are probably involved in the control of MeJA-induced isoflavonoid productions.

### 2.6. Prediction of CsbHLHs Involved in the Regulation of (Iso)Flavonoid Biosynthesis

**Figure 6:**
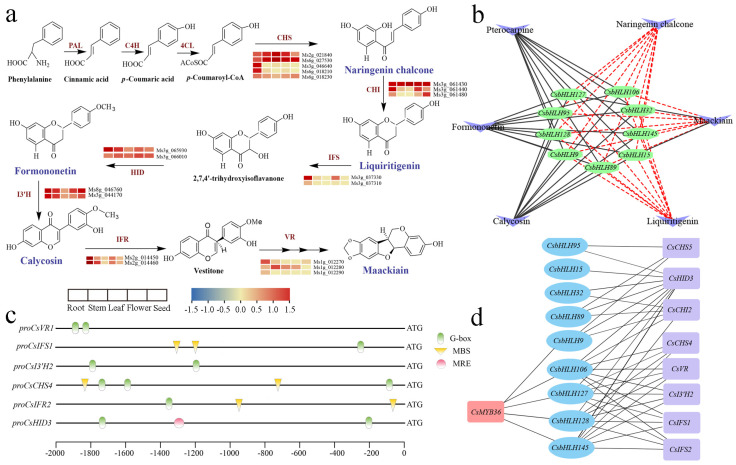
The anticipated regulatory network for the biosynthesis of isoflavonoids in *C. speciosa*. (**a**) A condensed illustration of the biosynthetic pathway of isoflavonoids. PAL, C4H, 4CL, CHS, CHI, IFS, HID, I’3H, IFR, and VR represent genes encoding phenylalanine ammonia lyase, cinnamic acid 4-hydroxylase, 4-coumarate-CoA ligase, chalcone synthase, chalcone isomerase, 2-hydroxyisoflavanone synthase, 2-hydroxyisoflavanone dehydratase, isoflavone 3′-hydroxylases, isoflavone reductase, and vestitone reductase, respectively. The gene expression levels in different plant parts are denoted by cubes arranged in the order of root, stem, leaf, flower, and seed, with the magnitude shown as log10 (FPKM). Change in color from blue to red represents low to high expression level. (**b**) The predicted regulatory network of the nine *CsbHLH* genes and six metabolites. (**c**) Analysis of promoters associated with genes involved in isoflavonoid biosynthesis. 2000 bp upstream of the initiation codon promoter sequences were analyzed for each gene. DNA binding sites were identified using PlantPAN 3.0. (**d**) The predicted regulatory network among *CsMYB36*, the nine *CsbHLH* genes, and eight structural genes involved in the (iso)flavonoid biosynthetic pathway.

Nineteen transcripts encoding structural genes essential for isoflavonoid biosynthesis were identified within the Meblack modules. These transcripts exhibited specific up-regulation in roots compared to other tissues, such as *CsVR1*, *CsIFS1*, *CsI3′H2*, *CsCHS4*, *CsIFR2*, and *CsHID3* (Figure 6a and Supplementary Table S6). Furthermore, a comprehensive set of 185 flavonoid compounds was determined through the application of ultra-high-performance liquid chromatography–tandem mass spectrometry technology, including 67 flavonols, 48 flavonoids, 21 isoflavones, 12 dihydroflavones, 14 tannins, 12 chalcones, 8 proanthocyanidins, and 3 dihydroflavonols. Among them, six compounds (pterocarpin, naringenin chalcone, formononetin, maackiain, calycosin, and liquiritigenin) were specially accumulated in roots (Figure S3 and Supplementary Table S7). Similar to the metabolomics data, the contents of the indicative compounds formononetin and maackiain were both at the highest levels in roots (Figure S1b). Three compounds (pterocarpin, formononetin, and calycosin) had a positive relation with the nine candidate *CsbHLH* genes (correlation coefficient > 0), while the other three compounds had a negative relation with them (correlation coefficient < 0) (Figure 6b).

The putative *cis*-regulatory elements of the six isoflavonoid-associated structural genes were investigated by extracting their 2000 bp upstream promoter region sequences (Figure 6c). The results showed that all of them contained G-box (5′-CACGTG-3′), which is mostly characterized by binding to bHLH proteins. The MBS and MRE motifs associated with MYB binding were also detected within the 2000 bp upstream promoter region sequences of *CsIFS1*, *CsCHS4*, and *CsHID3*. *CsMYB36* (Ms6g_048740) was found to have the capability to interact with the promoter regions of *CsIFS1* and *CsI3′H1* in *C. speciosa* [28]. Given the crucial regulatory function of bHLH TFs in isoflavonoid biosynthesis by forming protein complexes with MYB proteins to modulate secondary metabolism [34], we furtherly constructed the regulation network of *CsbHLHs*, *CsMYB36*, and structural genes in this study. As shown in Figure 6d, five *CsbHLHs* (*CsbHLH9*, *CsbHLH106*, *CsbHLH127*, *CsbHLH128*, and *CsbHLH145*) are expected to play significant roles in controlling the biosynthesis of isoflavonoids through forming the protein complex with *CsMYB36*. Additionally, the nine candidate *CsbHLH* genes showed a positive correlation with the expression of structural genes associated with the isoflavonoid biosynthesis pathway.

### 2.7. Verification of the Interaction Between CsMYB36 and CsbHLH9

**Figure 7:**
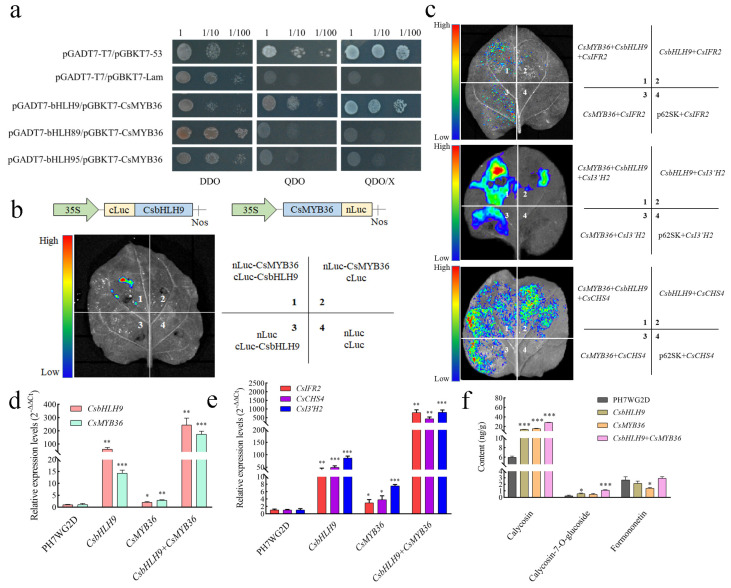
Interactions between CsMYB36 and CsbHLH9. (**a**) Yeast two-hybrid assays. DDO, SD/-Leu-Tr; QDO, SD/-Leu-Trp-His; QDO/X, SD/-Leu-Trp-His-Ade. (**b**) The interactions between CsMYB36 and bHLH9 by BiFC assays. (**c**) Dual-luciferase assays. nLuc and cLuc, the N- and C-terminal of firefly luciferase (Luc); nLuc-CsMYB36, CsMYB36 fused with the N-terminal of firefly luciferase; cLuc-bHLH9, bHLH9 fused with the C-terminal of firefly luciferase. (**d**) The relative expression profiles of CsMYB36, CsbHLH9 were analyzed using qRT-PCR in the Agrobacterium-mediated leaves. (**e**) qRT-PCR was used to detect the expression of genes encoded key enzymes in isoflavonoid biosynthesis pathway in Agrobacterium-mediated leaves. (**f**) The content of calycosin, calycosin-7-o-glucoside and formononetin in the Agrobacterium-mediated leaves. Significant variations were determined by the Student’s *t*-test (* *p* < 0.05, ** *p* < 0.01, *** *p* < 0.001).

Due to CsbHLHs (CsbHLH9, CsbHLH89, and CsbHLH95) belonging to subgroup III (d + e) had the motif 8 and being reported to positively regulate flavonoid biosynthesis [35,36], we selected them for further verification. A yeast two-hybrid (Y2H) assay was performed to assess the interaction between CsbHLHs (CsbHLH9, CsbHLH89, and CsbHLH95) and CsMYB36 (Figure 7a). The Y2H yeast cells in the positive control group (pGADT7-T7/pGBKT7-53) grow well on the QDO/X selective medium, while cells in the negative control group (pGADT7-T7/pGBKT7-Lam) cannot grow. In the experimental groups, Y2H yeast cells co-transformed with pGBKT7-CsbHLH9 and pGADT7-CsMYB36 demonstrated robust growth on the QDO/X selective medium, while other combinations did not exhibit growth, suggesting that only CsbHLH9 could interact with CsMYB36. We next used a bimolecular fluorescence complementation (BiFC) assay to confirm this interaction. The findings indicated that fluorescence emission was observed solely in the region co-expressing nLuc-CsMYB36 and cLuc-CsbHLH9 within *N. benthamiana* leaves, providing evidence that CsMYB36 physically interacted with CsbHLH9 in vivo (Figure 7b).

Dual-luciferase assays (LUC) results also revealed that *CsbHLH9* or *CsMYB36* separately activated the expression of *CsIFR2*, *CsI3′H2*, and *CsCHS4* promoters, but higher levels of activation luciferase activities were detected in the combination of *CsbHLH9* and *CsMYB36*. Co-infiltration of *N. benthamiana* leaves with *CsbHLH9* and *CsMYB36* also resulted in increased transcriptional activity of *CsIFR2*, *CsI3′H2*, and *CsCHS4* promoters compared to the infiltration of *CsbHLH9* or *CsMYB36* alone (Figure 7c). Collectively, these findings demonstrated that *CsbHLH9* interacted with *CsMYB36* to activate the promoter of *CsIFR2*, *CsI3′H2*, and *CsCHS4*.

### 2.8. Functional Validation of CsMYB36 and CsbHLH9

The *Agrobacterium*-mediated leaf disc infiltration method was performed in *C. speciosa* to validate the function of *CsbHLH9* and *CsMYB36*. Compared to the empty vector (pH7WG2D), the expression levels of *CsbHLH9* and *CsMYB36* were both up-regulated in the leave discs co-infiltrated with both *CsbHLH9* and *CsMYB36* after transfection for 6 d (Figure 7d). Co-infiltration of *C. speciosa* leaves with *CsbHLH9* and *CsMYB36* also resulted in significantly increased expression profiles of *CsIFR2*, *CsI3′H2*, and *CsCHS4* compared to the infiltration of both the empty vector and *CsbHLH9* or *CsMYB36* separately (Figure 7e). Higher expression levels of *CsbHLH9* and three structure genes related to isoflavonoid biosynthesis (*CsIFR2*, *CsI3′H2*, and *CsCHS4*) were detected in the co-infiltrated leave discs of CsbHLH9 than that of CsMYB36 *(*Figure 7d,e). Further, the content of three compounds (calycosin, calycosin-7-o-glucoside, and formononetin) was highly increased under the interaction effect of *CsbHLH9* and *CsMYB36 (*Figure 7f).

## 3. Discussion

The second largest transcription factor family bHLH, known for its remarkable diversity, plays a crucial role in various biological processes such as organogenesis, specialized metabolism, and responses to environmental stimuli [16,17,23]. Increasing reports demonstrated that bHLH could regulate flavonoid biosynthesis by forming MBW complexes (MYB-bHLH-WD40) in different plants [23,37]. In strawberry, the up-regulation of FaMYB5/FaMYB10 mediated by FaEGL3 (bHLH) facilitated flavonoid accumulation [38], while the R2R3-MYB transcriptional repressor *TgMYB4* combined with bHLH protein and negatively regulated anthocyanin biosynthesis in tulips [39]. Root-specific (iso)flavonoid accumulation is a major factor affecting the quality of *C. speciosa*, regulated by a network of multiple genes that constitute intricate regulatory mechanisms. Moreover, our previous study reported that *CsMYB36* (Ms6g_048740) has the capability to interact with the promoter regions of *CsIFS1* and *CsI3′H1* and was speculated to play an important role in regulating the isoflavonoid biosynthesis in the roots of *C. speciosa* [28]. However, the bHLH TFs responsible for isoflavonoid biosynthesis in *C. speciosa* have not yet been documented. Here, we identified 146 *bHLH* genes in *C. speciosa* by using the HMM profile of the HLH DNA-binding domain to blast the *C. speciosa* genome database and further verified them in the Pfam, SMART, and HHMER online databases. The majority (87.57%) of CsbHLH proteins were observed to be predominantly localized in the nucleus, a pattern consistent with their role as transcription factors (Supplementary Table S2). The *bHLH* gene families are characterized by their expansive nature, comprising 102 members in walnut (*Juglans regia* L.) [40], 159 in *Cyclocarya paliurus* [17], and 152 in tomato [10], which can be divided into 15–26 subfamilies relative to those of *Arabidopsis*.

Our phylogenetic analysis revealed that the CsbHLH proteins were grouped into 23 subfamilies, following the classification of the *Arabidopsis* bHLH family (Figure 1). To date, various investigations have been carried out to elucidate the roles and functions of *bHLH* genes in *Arabidopsis* [36]. Thus, the functions of the *CsbHLH* genes in *C. speciosa* were carried out to predict the homology between *AtbHLHs* and *CsbHLHs*. For instance, four members (*AtbHLH18*, *AtbHLH19*, *AtbHLH20*, and *AtbHLH25*) from subgroup IVa can be up-regulated by JA and suppress the transcription of the FIT (*AtbHLH29*) and *Ib bHLH* genes [41]. Members of the III (d + e) subfamily enhance plant resistance and regulate anthocyanin synthesis via the JA signaling pathway [35,42], whereas members of the IIIf subfamily have been demonstrated to participate in the biosynthesis of anthocyanins [43]. Notably, subgroups X, XIV, and XIII of the *Arabidopsis* classification did not include any CsbHLH proteins; similar results have been reported for walnut and *G. pentaphyllum*, which lack members in subgroups XIV and XIII, respectively, indicating that these species may have undergone adaptive evolution and modified the regulation of their shade response over the course of their evolutionary history [5,40]. Several highly conserved residues of the signature motif 1 appeared in all the CsbHLH protein sequences of *C. speciosa*, such as E (Glu), R (Arg), and L (Leu), and are necessary for the interaction with target DNA sequences (Supplementary Table S3). These findings are in agreement with previous reports [40,44]. The observed similarity of conserved motifs within a specific subgroup provided further evidence for the evolutionary classification of the *CsbHLH* gene family. Notably, motif 8 (orange) was only distributed in subgroup III (d + e), indicating that it could play a crucial role in the execution of the relevant regulatory functions.

The expansion of gene families typically arises from gene duplication events followed by subsequent diversification, which mainly consists of tandem repeats (intra-chromosome) and segmental duplication (extra-chromosome) [45]. The chromosomal localization results showed that the 146 *CsbHLH* genes exhibited non-uniform distribution across the chromosomes (Figure 3a). Fifty pairs of the 146 *CsbHLH* genes exhibited fragmental replication events (Figure 3b), comparable to the ratio documented in the *G. pentaphyllum* (31 out of 111) [5] and sorghum [*Sorghum bicolor* (L.) Moench] (42 out of 174) [46] bHLH families. Only a few tandem repeats were found in the above species, suggesting that fragmental replication may make a higher contribution to the amplification of the bHLH family. The promoter region of a gene harbors *cis*-regulatory elements, which are essential for regulating gene function [40]. In our study, the nine *CsbHLH* genes associated with isoflavonoids were identified to include two categories of *cis*-regulatory elements, specifically stress-responsive and hormone-responsive elements (such as ABA and MeJA) (Figure 5a), suggesting that isoflavonoid biosynthesis is influenced by various phytohormones and changing environmental conditions. The CGTCA motif and TGACG motif were identified as the predominant MeJA-responsive elements [5]. Interestingly, four *CsbHLH* genes (*CsbHLH9*, *CsbHLH32*, *CsbHLH89*, and *CsbHLH95*) did not contain the MeJA-responsive elements, but they were induced under MeJA treatment by qRT-PCR analysis (Figure 5c), which were in accordance with *GpbHLH15* and *GpbHLH25* in *G. pentaphyllum* [5]. Moreover, six *CsbHLH* gene promoters contained the bHLH binding site (G-box), and four contained MBS, suggesting that these proteins may engage in interactions with each other or with MYB TFs to modulate developmental and physiological processes. MeJA has been proven to be an effective inducer that can cause the accumulation of isoflavonoids [47]. In our previous study, we found that the indicative compounds formononetin and maackiain were obviously increasingly induced by 200 μmol/L MeJA by activating the key enzyme genes in isoflavonoid biosynthesis in *C. speciosa*, such as *CHS2*, *HIDs*, *I3′Hs*, etc. [33]. In the current study, *CsbHLH9* positively regulated calycosin, calycosin-7-o-glucoside, and formononetin biosynthesis mainly via *CsIFR2*, *CsI3′H2*, and *CsCHS4* genes. It is one of the most significantly up-regulated bHLH genes induced by MeJA (Figure 5d), suggesting that it might function in isoflavonoid biosynthesis in a MeJA-induced manner.

Most CsbHLHs showed distinct expression patterns in five different tissues, suggesting their functions varied in the special tissue (Figure 4). The metabolomics results showed that 21 isoflavones were specially accumulated in roots (Figure S3 and Supplementary Table S7). Similar to the metabolomics data, the contents of the indicative compounds formononetin and maackiain were both at the highest levels in roots (Figure S1b). Nine *CsbHLH* genes exhibited notably high expression levels in roots and were identified as candidate genes associated with isoflavonoid biosynthesis (Supplementary Table S6), which had positive relationships with three metabolites (pterocarpin, formononetin, and calycosin) through the root-specific isoflavonoid regulatory network linking genes to metabolites. Six of nine *CsbHLHs* were also identified by transcriptomic analysis in our previous research [48], which were differentially expressed during the process of formononetin and maackiain accumulation, suggesting their potential roles in the regulation of isoflavonoid biosynthesis. Based on our previous studies [28,49], *CsMYB36* could bind to the promoters of structural genes in the isoflavonoid metabolic pathway and likely played a role in regulating isoflavonoid biosynthesis in *C. speciosa*. Different types of transcription factors can form complexes to regulate downstream target genes. For instance, SmMYB1 has been reported to form a complex with SmMYC2 to regulate SmCYP98A14 and induce phenolic acid accumulation [50]. In the present study, we identified that the CsMYB36 interacted with CsbHLH9 through the Y2H assays (Figure 7a). BiFC assays also verified the interaction between CsbHLH9 and CsMYB36 (Figure 7b). These results revealed that CsbHLH9 directly interacted with CsMYB36 to form a complex. Moreover, the LUC assay results demonstrated that the CsbHLH9 and CsMYB36 complex led to a rise in the transcriptional activation of *CsIFR2*, *CsI3′H2*, and *CsCHS4* (Figure 7c). Thus, we postulate that the CsbHLH9 and CsMYB36 complex positively regulates *CsIFR2*, *CsI3′H2*, and *CsCHS4* to promote isoflavonoid biosynthesis. In order to verify our hypothesis, we performed the transient transformation of CsbHLH9 and CsMYB36 in *C. speciosa* leaves. The results also showed that the CsbHLH9 and CsMYB36 complex promoted the expression profiles of *CsIFR2*, *CsI3′H2*, and *CsCHS4*, thereby facilitating the accumulation of calycosin, calycosin-7-o-glucoside, and formononetin (Figure 7e,f). Furthermore, we found that *CsbHLH9* and *CsMYB36* have different regulatory strengths on *CsIFR2*, *CsI3′H2* and *CsCHS4*, respectively. Based on these results, we speculate that *CsbHLH9 may have* played a role in isoflavonoid biosynthesis as a core transcription activator. However, how the CsbHLH9 and CsMYB36 work together to regulate isoflavonoid biosynthesis still need more exploration in the future.

## 4. Materials and Methods

### 4.1. Plant Materials and MeJA Treatment

The plant material employed in this investigation is a diploid medicinal plant, the genome of which was sequenced in a prior study conducted by our team [28]. Five distinct tissues from three-year-old plants of *C. speciosa*, namely roots, stems, leaves, flowers, and seeds (Figure S1a), were gathered for transcriptomic sequencing by the Illumina HiSeq platform. For MeJA treatment, three-month-old seedlings were sprayed with 200 μmol/L MeJA solution until dripped. Root samples were obtained from ten seedlings at 0 h before treatment, as well as at 6, 24, and 72 h post-treatment.

### 4.2. Identification and Annotation of CsbHLH Proteins

The genome sequences of *C. speciosa* were obtained from the *C. speciosa* genome database. The HLH-conserved signature domain (PF00010) Hidden Markov Model (HMMER v3.3.2) was acquired from the Pfam online tool (http://pfam.xfam.org/, accessed on 15 March 2022) and subsequently applied to query the genome sequences of *C. speciosa*. Then, the sequences (*E* value < 10^−5^) were extracted using bidirectional BLASTP comparisons. Candidate proteins harboring the bHLH domain were subsequently validated using the NCBI Conserved Domains Database (CDD) and the Simple Modular Architecture Research Tool (SMART). Redundancies and partial sequences were eliminated. The bHLH family genes of *Arabidopsis thaliana* were sourced from the *Arabidopsis* database (TAIR; https://www.arabidopsis.org/, accessed on 27 March 2022).

### 4.3. Subcellular Localization and Properties Prediction

Following the sequential reassignment of all the designated *CsbHLHs* in accordance with their chromosomal arrangement, an assessment of their physical and chemical characteristics was conducted utilizing the ExPASy ProtParam web tool (https://web.expasy.org/protparam/, accessed on 2 May 2022), including molecular weight (MW), amino acid length, predicted pI, instability index (II), and grand average of hydropathicity (GRAVY). Subcellular localization was predicted by online Predictprotien software (https://predictprotein.org/, accessed on 12 May 2022).

### 4.4. Phylogenetic Analysis and Multiple Sequence Alignment

Multiple sequence alignments of CsbHLHs from *C. speciosa* and AtbHLH proteins from *Arabidopsis thaliana* were performed using Clustal X (v2.0) with standard settings, as described in a previous study [51]. A maximum likelihood (ML) tree of CsbHLH proteins was constructed with MEGA 6.0 software (version 6.0, Mega Limited, Auckland, New Zealand) with 1000 bootstraps [52]. For visualization, customization, and annotation of the phylogenetic tree, the Interactive Tree of Life (iTOL) version 5 web-based application was employed [53]. The subfamily of CsbHLH proteins was classified based on the evolutionary relationships of AtbHLH proteins.

### 4.5. Conserved Motif and Gene Structure Analysis

The conserved motifs were analyzed using the online MEME (v5.1.1) with default settings, except that a limit of 10 motifs was chosen as the maximum number for identification. For the exon/intron structure map of the *CsbHLHs*, the utilization of the TBtools software (v0987663, CJ-Chen, Guangzhou, China) [54] was grounded on the structural details provided in the GFF annotation files of the *C. speciosa* genome database. Additionally, MEGA6.0 software was used for building an ML phylogenetic tree of 146 CsbHLHs. Finally, the phylogenetic tree, motifs, domains, and exon/intron structure were visualized by TBtools software.

### 4.6. Chromosomal Locations and Collinearity

The localization of individual *CsbHLH* genes was determined by aligning *bHLH* gene sequences with chromosome survey sequences of *C. speciosa*, with their respective positions annotated on the 8 chromosomes through the utilization of BLAST algorithms. The chromosomal positioning outcomes were visually represented utilizing the MG2C tool (http://mg2c.iask.in/mg2c_v2.0/, accessed on 20 September 2022), as described in [55]. The assessment of collinearity regions and gene duplication events within the *bHLH* gene family between *C. speciosa* and *A. thaliana* was assessed, as described previously [5]. Utilizing Tbtools software, the non-synonymous replacement rate (Ka) and synonymous replacement rate (Ks) for each duplicated *bHLH* gene pair were computed. The Ka/Ks ratio was subsequently employed to assess the environmental selection pressure.

### 4.7. Metabolomics and the Indicative Compound Contents Measurement

For flavonoid metabolomics, five different tissues were freeze-dried and extracted according to the instructions of METWARE (Wuhan, China). Sample extracts were analyzed using an LC-ESI-MS/MS system [UPLC, SHIMADZU Nexera X2; MS, Applied Biosystems 4500 Q TRAP (ThermoFisher, Waltham, MA, USA); MS, ESI-Q TRAP]. Chromatographic separation was performed on an Agilent SB-C18 column (1.8 µm, 2.1 mm × 100 mm) using mobile phase A (0.1% formic acid in pure water) and mobile phase B (0.1% acetic acid in acetonitrile). The elution profile was used as follows: 95:5 v(A)/v(B) at 0 min, 5:95 v(A)/v(B) at 9.0 min, 5:95 v(A)/v(B) at 1.0 min, 95:5 v(A)/v(B) at 1.10 min, and 95:5 v(A)/v(B) at 1.9 min. The flow velocity was maintained at 0.35 mL/min, and the injection volume was 4 µL. Mass data acquisition was performed in electrospray ionization positive/negative mode using the following parameters: ion source, turbo spray; source temperature 550 °C; ion spray voltage (IS) 5500 V (positive ion mode)/−4500 V (negative ion mode); ion source gas I (GSI), gas II (GSII), curtain gas (CUR) were set at 50, 60, and 25.0 psi, respectively; the collision-activated dissociation (CAD) was high. Instrument tuning and mass calibration were performed with 10 and 100 μmol/L polypropylene glycol solutions in triple quadrupole and linear ion trap modes, respectively. Declustering potential (DP) and collision energy (CE) for individual multiple reaction monitoring (MRM) transitions were performed with specific DP and CE optimization. A specific set of MRM transitions was monitored for each period based on the metabolites eluted within this period. The metabolites were identified by comparing the m/z values, the retention time (RT), and the fragmentation patterns with the standards in a self-compiled database (MetWare). Significantly changed metabolites (SCMs) were filtered according to |Log2 (fold change)| ≥  1, *p*-value  <  0.05.

Extraction and analysis of the indicative compounds formononetin and maackiain were performed, as described previously [48].

### 4.8. Identification of Co-Expression Modules

In our previous study, the fragments per kilobase of transcript per million fragments mapped (FPKM) values of five distinct tissues (roots, stems, leaves, flowers, and seeds) were obtained from the NCBI database under the accession number PRJNA720501 [28]. The expression values of *CsbHLH* genes were displayed using Tbtools software according to their FPKM values. The weighted gene co-expression network analysis (WGCNA) package was applied for conducting co-expression analysis.

### 4.9. Expression Confirmation by qRT-PCR

Extraction of total RNA from various tissue types was performed using a reference RNA extraction kit (Super Plant Genomic DNA Kit (Ploysaccharides & Ployphenolics-rich), Beijing Tiangen Company, Beijing, China).and reverse transcription was conducted following previously reported procedures [48]. qRT-PCR was carried out using LightCycler 96 System (BIO-RAD C1000 Touch ^TM^ Thhermal Cycler, Singapore, Singapore), and the reaction conditions followed our previous study [49]. The *CsGAPDH* gene was utilized as an internal control across various tissue types and various *Agrobacterium* mediated in *C. speciosa* leaves [56]. The *CsACTIN* gene was employed as an internal control in the up-regulation process induced by methyl jasmonate. The 2^−ΔΔCt^ analysis method was used to assess the relative expression levels of genes. The primers employed in this study are detailed in Supplementary Table S1.

### 4.10. Dual Luciferase (LUC) Assays

The DNA binding sites within a 2000 bp region upstream of the initiation codon of *CsbHLH* genes and promoter sequences were predicted utilizing the PlantPAN 3.0 database (http://plantpan.itps.ncku.edu.tw/, accessed on 20 September 2022). For the promoter cloning, their promoter sequences were amplified and inserted separately into the vector pGreenII0800 as reporters. The coding sequences (CDS) of *CsbHLH9* and *CsMYB36* were individually cloned and inserted into the p62SK vector as effectors. Each vector was independently transformed into the *Agrobacterium* GV3101 strain (GV3101 Chemically Competent Cell, Shanghai Weidi Biotechnology, Shanghai, China) cultured in 100 mg/mL kanamycin-resistant medium at 28 °C. The *Agrobacteriums* were then centrifuged into pellets and resuspended in a buffer solution containing 10 mmol/L MES, 10 mmol/L MgCl_2_, and 15 µL 100 mmol/L acetosyringone at pH 5.6 at an optical density of 0.8 at 600 nm. Solutions of regulators, reporter, and P19 were combined in a ratio of 3.5:2:1 (*v*/*v*). The *Agrobacteriums* were then incubated for 3 h at room temperature in the dark. The resuspensions were co-injected into the leaves of *N. benthamiana*. The leaves co-injected by the empty vector p62SK were used as the control. After injection for 48 h, the infiltrated leaves were brushed with D-Luciferin, potassium salt (Goldbio, Fairview, TX, USA), and the fluorescence detection was conducted utilizing a fluorescence imaging system (IVIS Lumina LT, Americas, USA). The primer sequences are documented in Supplementary Table S1.

### 4.11. Bimolecular Fluorescence Complementation (BiFC) Assays

Bimolecular fluorescence complementation (BiFC) assays were performed following a previously described protocol [57]. Full-length coding sequences of CsMYB36 and CsbHLH9 were cloned into JW771 and JW772, respectively. The *Agrobacteriums* and P19 were combined in a ratio of 4:1 (*v*/*v*). Four different mixtures, CsMYB36-nLuc + cLuc-CsbHLH9, CsMYB36-nLuc + cLuc, nLuc +cLuc-CsbHLH9, and nLuc + cLuc, were co-injected into four different parts of *N. benthamiana* leaves. Images were captured using a fluorescence imaging system (IVIS Lumina LT, America, USA) 48 h after transfection. The primers are listed in Supplementary Table S1.

### 4.12. Yeast Two-Hybrid (Y2H) Assays

In the Y2H assay, the *CsMYB36* gene was connected to the pGBKT7 vector, and three *CsbHLH* genes (*CsbHLH9*, *CsbHLH89*, and *CsbHLH95*) were connected separately to the pGADT7 vector, respectively. The primers are presented in Supplementary Table S1. The experimental group consisted of the yeast Y2H co-transformed with pGBKT7-CsMYB36 and pGADT7-CsbHLHs. The empty vector (pGBKT7-Lam and pGADT7-T7) and the recombined vector (pGBKT7-53 and pGADT7-T7) were also co-transformed as negative and positive controls, respectively. Following the selection of monoclonal strains on SD/-Leu-Trp (DDO, Coolaber, Beijing, China) solid medium, their growth was evaluated on SD/-Leu-Trp-His-Ade (QDO, Coolaber, Beijing, China) solid medium in the presence or absence of X-α-gal (5-Bromo-4-chloro-3-indolyl β-D-galactopyranoside, Coolaber, Beijing, China). The yeast strains were cultured in a 30 °C incubator for a period of 3–4 days, during which their growth was monitored.

### 4.13. Transient Transformation of CsMYB36 and CsbHLH9 in C. Speciosa Leaves

Full-Length coding sequences of *CsMYB36* and *CsbHLH9* were cloned into pH7WG2D using the gateway method, respectively. Four different groups, pH7WG2D, pH7WG2D-CsbHLH9, pH7WG2D-CsMYB36, and pH7WG2D-CsMYB36 + pH7WG2D-CsbHLH9, were co-injected into *C. speciosa* leaves using previously described methods. The leaves were collected and ground to a powder after 6 d of transfection. The primers are listed in Supplementary Table S1. A total of 0.4 g of *C. speciosa* leaves powder was weighed for isoflavones extraction with 80% methanol solution (4:1, *v*/*v*) with 30 mL solution added, and ultrasonic extraction was performed for 1 h, then filtered with a 0.22 μM microporous membrane. The contents of three compounds (calycosin, calycosin-7-o-glucoside, and formononetin) were determined by the HPLC (Shimadzu LC-40D, Kyoto, Japan) method, which was performed according to our described previously [48]. The formononetin was detected at 310 nm, but the detection wavelength of others was 245 nm. Finally, the content was calculated by substituting it into the standard curve. All measurements were carried out in three biological replicates.

### 4.14. Statistical Analyses

Significance was tested by the Student’s *t*-test, were performed using IBM SPSS Statistics software (version24.0, IBM, New York, NY, USA). Graphpad prism software (version 8.0.2, GraphPad Software, San Diego, CA, USA) was used to create figures. “*”, “**”, and “***” in figures were considered statistically significant at *p* < 0.05, *p* < 0.01, and *p* < 0.001, respectively.

## 5. Conclusions

This study conducted a comprehensive analysis across the entire genome to investigate the *bHLH* gene family in *C. speciosa*, with a focus on their involvement in regulating isoflavonoid biosynthesis. Nine candidate *CsbHLH* genes were identified as potential regulators of isoflavonoid biosynthesis through a combination of gene-to-metabolite correlation analysis and WGCNA. Analysis of *cis*-regulatory elements and expression profiles revealed distinct patterns among these candidate *CsbHLH* genes, indicating their varied functions. Interactions between three CsbHLHs (CsbHLH9, CsbHLH89, and CsbHLH95) and CsMYB36 (a MYB transcription factor that has been reported to participate in isoflavonoid biosynthesis in our previous study) were identified via Y2H, LUC, and BiFC assays. Importantly, we found CsbHLH9 acts as a transcriptional activator through its interaction with CsMYB36 and binding to the promoters of isoflavonoid biosynthesis genes in a MeJA-induced manner, such as *CsIFR2*, *CsI3′H2*, and *CsCHS4* to promote isoflavonoids (calycosin, calycosin-7-o-glucoside, and formononetin) accumulation. To conclude, our findings provide an invaluable understanding of the roles of the MBW complex in regulating isoflavonoid biosynthesis in *C. speciosa*, facilitating the formulation of effective strategies for enhancing isoflavonoid genetic improvement.

## Data Availability

Data will be made available upon request. The transcriptome sequencing data have been deposited in NCBI under accession code PRJNA720501.

## Supplementary Materials

The following supporting information can be downloaded at https://www.mdpi.com/article/10.3390/ijms252211900/s1.

## References

[1] Song M., Wang H., Wang Z., Huang H., Chen S., Ma H. (2021). Genome-wide characterization and analysis of bHLH transcription factors related to anthocyanin biosynthesis in fig (*Ficus carica* L.). Front. Plant Sci..

[2] Toledo-Ortiz G., Huq E., Quail P.H. (2003). The *Arabidopsis* basic/helix-loop-helix transcription factor family. Plant Cell.

[3] Heim M.A., Jakoby M., Werber M., Martin C., Weisshaar B., Bailey P.C. (2003). The basic helix-loop-helix transcription factor family in plants: A genome-wide study of protein structure and functional diversity. Mol. Biol. Evol..

[4] Li X., Duan X., Jiang H., Sun Y., Tang Y., Yuan Z., Guo J., Liang W., Chen L., Yin J. (2006). Genome-wide analysis of basic/helix-loop-helix transcription factor family in rice and *Arabidopsis*. Plant Physiol..

[5] Qin Y., Li J., Chen J., Yao S., Li L., Huang R., Tan Y., Ming R., Huang D. (2024). Genome-wide characterization of the *bHLH* gene family in *Gynostemma pentaphyllum* reveals its potential role in the regulation of gypenoside biosynthesis. BMC Plant Biol..

[6] Grotewold E., Sainz M.B., Tagliani L., Hernandez J.M., Bowen B., Chandler V.L. (2000). Identification of the residues in the Myb domain of maize C1 that specify the interaction with the bHLH cofactor R. Proc. Natl. Acad. Sci. USA.

[7] Massari M.E., Murre C. (2000). Helix-loop-helix proteins: Regulators of transcription in eucaryotic organisms. Mol. Cell Biol..

[8] Pires N., Dolan L. (2010). Origin and diversification of basic-helix-loop-helix proteins in plants. Mol. Biol. Evol..

[9] Carretero-Paulet L., Galstyan A., Roig-Villanova I., Martínez-García J.F., Bilbao-Castro J.R., Robertson D.L. (2010). Genome-wide classification and evolutionary analysis of the bHLH family of transcription factors in *Arabidopsis*, poplar, rice, moss, and algae. Plant Physiol..

[10] Wang J., Hu Z., Zhao T., Yang Y., Chen T., Yang M., Yu W., Zhang B. (2015). Genome-wide analysis of bHLH transcription factor and involvement in the infection by yellow leaf curl virus in tomato (*Solanum lycopersicum*). BMC Genom..

[11] Wang Z., Cui Y., Vainstein A., Chen S., Ma H. (2017). Regulation of fig (*Ficus carica* L.) fruit color: Metabolomic and transcriptomic analyses of the flavonoid biosynthetic pathway. Front. Plant Sci..

[12] Strygina K.V., Khlestkina E.K. (2019). Myc-like transcriptional factors in wheat: Structural and functional organization of the subfamily I members. BMC Plant Biol..

[13] Bian S., Tian T., Ding Y., Yan N., Wang C., Fang N., Liu Y., Zhang Z., Zhang H. (2021). bHLH transcription factor NtMYC2a regulates carbohydrate metabolism during the pollen development of tobacco (*Nicotiana tabacum* L. cv. TN90). Plants.

[14] Chang G., Ma J., Wang S., Tang M., Zhang B., Ma Y., Li L., Sun G., Dong S., Liu Y. (2023). Liverwort bHLH transcription factors and the origin of stomata in plants. Curr. Biol..

[15] Lu R., Zhang J., Liu D., Wei Y.L., Wang Y., Li X.B. (2018). Characterization of bHLH/HLH genes that are involved in brassinosteroid (BR) signaling in fiber development of cotton (*Gossypium hirsutum*). BMC Plant Biol..

[16] Groszmann M., Paicu T., Smyth D.R. (2008). Functional domains of SPATULA, a bHLH transcription factor involved in carpel and fruit development in *Arabidopsis*. Plant J..

[17] Zhang Z., Fang J., Zhang L., Jin H., Fang S. (2023). Genome-wide identification of bHLH transcription factors and their response to salt stress in *Cyclocarya paliurus*. Front. Plant Sci..

[18] Zhao Q., Ren Y.R., Wang Q.J., Yao Y.X., You C.X., Hao Y.J. (2016). Overexpression of *MdbHLH104* gene enhances the tolerance to iron deficiency in apple. Plant Biotechnol. J..

[19] Qiu J.R., Huang Z., Xiang X.Y., Xu W.X., Wang J.T., Chen J., Song L., Xiao Y., Li X., Ma J. (2020). MfbHLH38, a *Myrothamnus flabellifolia* bHLH transcription factor, confers tolerance to drought and salinity stresses in *Arabidopsis*. BMC Plant Biol..

[20] Liao W., Cai J., Xu H., Wang Y., Cao Y., Ruan M., Chen S., Peng M. (2023). The transcription factor *MebHLH18* in cassava functions in decreasing low temperature-induced leaf abscission to promote low-temperature tolerance. Front. Plant Sci..

[21] Xu W., Dubos C., Lepiniec L. (2015). Transcriptional control of flavonoid biosynthesis by MYB-bHLH-WDR complexes. Trends Plant Sci..

[22] Bai H., Song Z., Zhang Y., Li Z., Wang Y., Liu X., Ma J., Quan J., Wu X., Liu M. (2020). The bHLH transcription factor PPLS1 regulates the color of pulvinus and leaf sheath in foxtail millet (*Setaria italica*). Theor. Appl. Genet..

[23] Jiang L., Yue M., Liu Y., Zhang N., Lin Y., Zhang Y., Wang Y., Li M., Luo Y., Zhang Y. (2023). A novel R2R3-MYB transcription factor FaMYB5 positively regulates anthocyanin and proanthocyanidin biosynthesis in cultivated strawberries (*Fragaria  ×  ananassa*). Plant Biotechnol. J..

[24] Zhang J., Wang J., Wang Y., Chen M., Shi X., Zhou X., Zhang Z. (2022). Phytochemistry and antioxidant activities of the rhizome and radix of *Millettia speciosa* based on UHPLC-Q-Exactive Orbitrap-MS. Molecules.

[25] Lam V.Q., Anh H., Quan N.V., Xuan T.D., Hanamura I., Uchino K., Karnan S., Takami A. (2022). Cytotoxicity of *Callerya speciosa* fractions against myeloma and lymphoma cell lines. Molecules.

[26] Wang M., Zhang M., Yang Q., Wang Q., Ma B., Li Z., Cheng W., Tang H., Feng S., Wang Z. (2021). Metabolomic profiling of *M. speciosa* champ at different growth stages. Food Chem..

[27] Zhang M., Cui C., Lin Y., Cai J. (2021). Ameliorating effect on glycolipid metabolism and chemical profile of *Millettia speciosa* champ. extract. J. Ethnopharmacol..

[28] Huang D., Yu L., Ming R., Tan X., Li L., Huang R., Tan Y., Yao S. (2023). A chromosome-level genome assembly of *Callerya speciosa* sheds new light on the biosynthesis of root-specific isoflavonoids. Ind. Crops Prod..

[29] Doerge R.W. (2002). Mapping and analysis of quantitative trait loci in experimental populations. Nat. Rev. Genet..

[30] Zernova O.V., Lygin A.V., Widholm J.M., Lozovaya V.V. (2009). Modification of isoflavones in soybean seeds via expression of multiple phenolic biosynthetic genes. Plant Physiol. Biochem..

[31] Huang W., Lv H., Wang Y. (2017). Functional characterization of a novel R2R3-MYB transcription factor modulating the flavonoid biosynthetic pathway from *Epimedium sagittatum*. Front. Plant Sci..

[32] Shen G., Wu R., Xia Y., Pang Y. (2021). Identification of Transcription Factor Genes and Functional Characterization of *PlMYB1* From *Pueraria lobata*. Front. Plant Sci..

[33] Yu L., Chen L., Qin C., Ming R., Huang D., Huang R., Yao S. (2023). Effects of methyl jasmonateon the accumulation of active components and related gene expressions in *Callerya speciosa*. Chin. Med. Mat..

[34] Sohn S.I., Pandian S., Oh Y.J., Kang H.J., Cho W.S., Cho Y.S. (2021). Metabolic engineering of isoflavones: An updated overview. Front. Plant Sci..

[35] Song S., Qi T., Fan M., Zhang X., Gao H., Huang H., Wu D., Guo H., Xie D. (2013). The bHLH subgroup IIId factors negatively regulate jasmonate-mediated plant defense and development. PLoS Genet..

[36] Hao Y., Zong X., Ren P., Qian Y., Fu A. (2021). Basic helix-loop-helix (bHLH) transcription factors regulate a wide range of functions in *Arabidopsis*. Int. J. Mol. Sci..

[37] Xu P., Wu L., Cao M., Ma C., Xiao K., Li Y., Lian H. (2021). Identification of MBW complex components implicated in the biosynthesis of flavonoids in woodland strawberry. Front. Plant Sci..

[38] Yue M., Jiang L., Zhang N., Zhang L., Liu Y., Lin Y., Zhang Y., Luo Y., Zhang Y., Wang Y. (2023). Regulation of flavonoids in strawberry fruits by FaMYB5/FaMYB10 dominated MYB-bHLH-WD40 ternary complexes. Front. Plant Sci..

[39] Hu X., Liang Z., Sun T., Huang L., Wang Y., Chan Z., Xiang L. (2024). The R2R3-MYB transcriptional repressor *TgMYB4* negatively regulates anthocyanin biosynthesis in tulips (*Tulipa gesneriana* L.). Int. J. Mol. Sci..

[40] Zhao W., Liu Y., Li L., Meng H., Yang Y., Dong Z., Wang L., Wu G. (2021). Genome-wide identification and characterization of bHLH transcription factors related to anthocyanin biosynthesis in red walnut (*Juglans regia* L.). Front. Genet..

[41] Cui Y., Chen C.L., Cui M., Zhou W.J., Wu H.L., Ling H.Q. (2018). Four IVa bHLH transcription factors are novel interactors of FIT and mediate JA inhibition of iron uptake in *Arabidopsis*. Mol. Plant.

[42] Liu X.J., An X.H., Liu X., Hu D.G., Wang X.F., You C.X., Hao Y.J. (2017). MdSnRK1.1 interacts with MdJAZ18 to regulate sucrose-induced anthocyanin and proanthocyanidin accumulation in apple. J. Exp. Bot..

[43] Gonzalez A., Zhao M., Leavitt J.M., Lloyd A.M. (2008). Regulation of the anthocyanin biosynthetic pathway by the TTG1/bHLH/Myb transcriptional complex in *Arabidopsis* seedlings. Plant J..

[44] Yingqi H., Ahmad N., Yuanyuan T., Jianyu L., Liyan W., Gang W., Xiuming L., Yuanyuan D., Fawei W., Weican L. (2019). Genome-wide identification, expression analysis, and subcellular localization of *Carthamus tinctorius* bHLH transcription factors. Int. J. Mol. Sci..

[45] Vision T.J., Brown D.G., Tanksley S.D. (2000). The origins of genomic duplications in *Arabidopsis*. Science.

[46] Fan Y., Yang H., Lai D., He A., Xue G., Feng L., Chen L., Cheng X.B., Ruan J., Yan J. (2021). Genome-wide identification and expression analysis of the bHLH transcription factor family and its response to abiotic stress in sorghum [*Sorghum bicolor* (L.) Moench]. BMC Genom..

[47] Wu Z., Zeng W., Li C., Wang J., Shang X., Xiao L., Cao S., Zhang Y., Xu S., Yan H. (2023). Genome-wide identification and expression pattern analysis of R2R3-MYB transcription factor gene family involved in puerarin biosynthesis and response to hormone in *Pueraria* lobata var. thomsonii. BMC Plant Biol..

[48] Yao S., Lan Z., Huang R., Tan Y., Huang D., Gu J., Pan C. (2021). Hormonal and transcriptional analyses provides new insights into the molecular mechanisms underlying root thickening and isoflavonoid biosynthesis in *Callerya speciosa* (Champ. ex Benth.) Schot. Sci. Rep..

[49] Yu L., Huang D., Gu J., Pan D., Tan Y., Huang R., Yao S. (2021). Identification of isoflavonoid biosynthesis-related R2R3-MYB transcription factors in *Callerya speciosa* (Champ. ex Benth.) Schot using transcriptome-based gene coexpression analysis. Int. J. Genom..

[50] Zhou W., Shi M., Deng C., Lu S., Huang F., Wang Y., Kai G. (2021). The methyl jasmonate-responsive transcription factor SmMYB1 promotes phenolic acid biosynthesis in *Salvia miltiorrhiza*. Hortic. Res..

[51] Thompson J.D., Gibson T.J., Plewniak F., Jeanmougin F., Higgins D.G. (1997). The CLUSTAL_X windows interface: Flexible strategies for multiple sequence alignment aided by quality analysis tools. Nucleic Acids Res..

[52] Tamura K., Stecher G., Peterson D., Filipski A., Kumar S. (2013). MEGA6: Molecular evolutionary genetics analysis version 6.0. Mol. Biol. Evol..

[53] Letunic I., Bork P. (2021). Interactive tree of life (iTOL) v5: An online tool for phylogenetic tree display and annotation. Nucleic Acids Res..

[54] Chen C., Chen H., Zhang Y., Thomas H.R., Frank M.H., He Y., Xia R. (2020). TBtools: An integrative toolkit developed for interactive analyses of big biological data. Mol. Plant.

[55] Chao J., Li Z., Sun Y., Aluko O.O., Wu X., Wang Q., Liu G. (2021). MG2C: A user-friendly online tool for drawing genetic maps. Mol. Hortic..

[56] Yu L., Ming R., Huang D., Qin C., Li L., Tan Y., Huang R., Yao S. (2022). Selection and validation of suitable reference genes for gene expression studies in *Callerya speciosa* (Champ. ex Benth.) Schot under different experimental conditions. ACS Agric. Sci. Technol..

[57] He J., Xu Y., Huang D., Fu J., Liu Z., Wang L., Zhang Y., Xu R., Li L., Deng X. (2022). TRIPTYCHON-LIKE regulates aspects of both fruit flavor and color in citrus. J. Exp. Bot..

