# Supplementary material for: Genome-Wide Identification of the bHLH Gene Family in Callerya speciosa Reveals Its Potential Role in the Regulation of Isoflavonoid Biosynthesis
Source: Int J Mol Sci. 2024 Nov 6;25(22):11900. doi: 10.3390/ijms252211900 (PMC11593548; doi:10.3390/ijms252211900)
Supplement: Supplementary file 1 [file ijms-25-11900-s001.zip › Supplementary Figures.pdf]

Genome-Wide Identification of the bHLH Gene Family in *Callerya speciosa* Reveals Its Potential Role in the Regulation of Isoflavonoid Biosynthesis

Liuping Chen [1,†], Xiaoming Tan [1,2,†], Ruhong Ming [1,2], Ding Huang [1,2], Yong Tan [1,2], Liangbo Li [1], Rongshao Huang [1] and Shaochang Yao [1,2,*]

[1] College of Pharmacy, Guangxi University of Chinese Medicine, Nanning 530200, China

[2] Guangxi Key Laboratory of Zhuang and Yao Ethnic Medicine, Guangxi University of Chinese Medicine, Nanning 530200, China

* Correspondence: yaosc@gxtcmu.edu.cn; Tel.: +86-0771-3941063

† These authors contributed equally to this work.

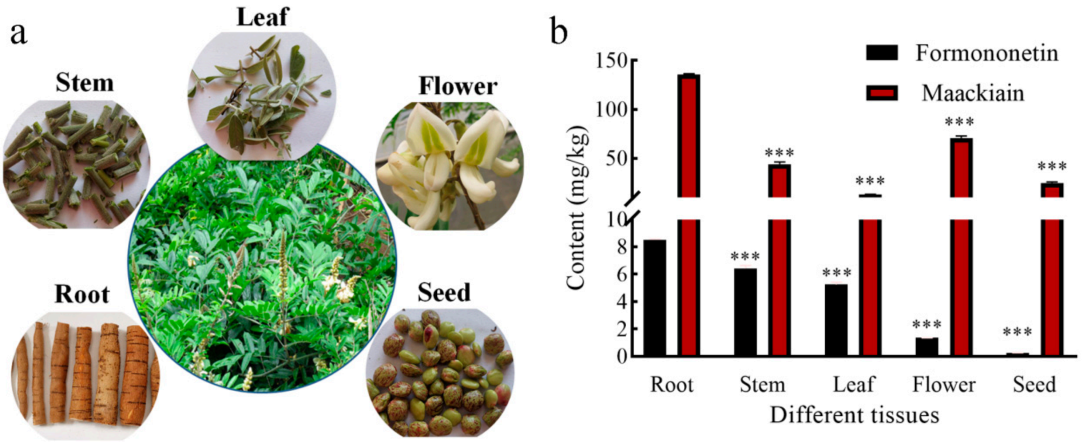

Figure S1. The phenotypic features (a) and indicative compound contents (b) of five different tissues of *C. speciosa*.

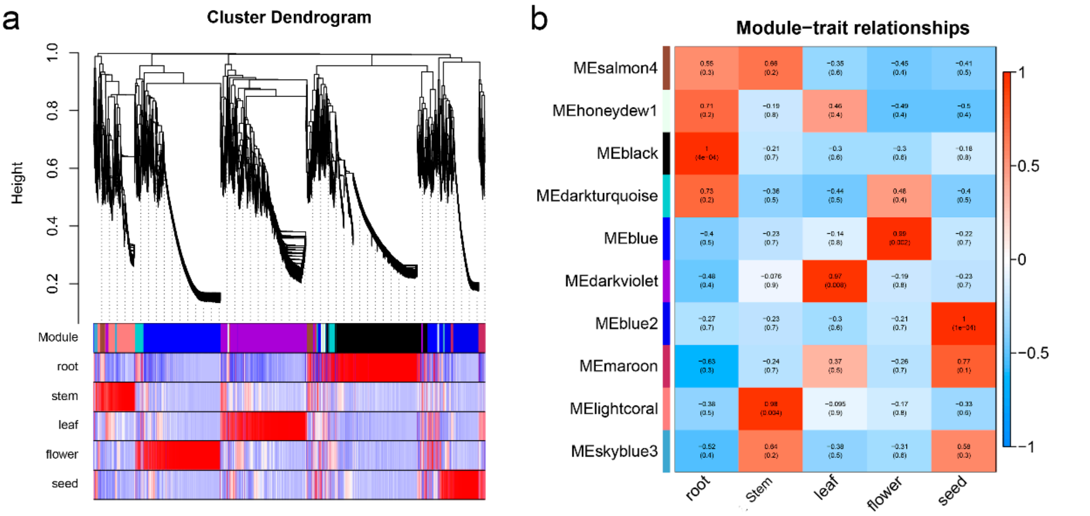

Figure S2. Co-expression network showing isoflavonoid-related module identified by WGCNA in *C. speciosa*. a. Hierarchical clustering tree (cluster dendrogram) showing ten modules of co-expressed genes with color annotation. b. Correlation between each module and five different tissues. Pearson correlation coefficients and *p* values (in brackets) are indicated in the grid where modules and traits intersect. A low to high degree of Pearson correlation coefficient between a specific module and the tissue is indicated by a change in color from blue to red.

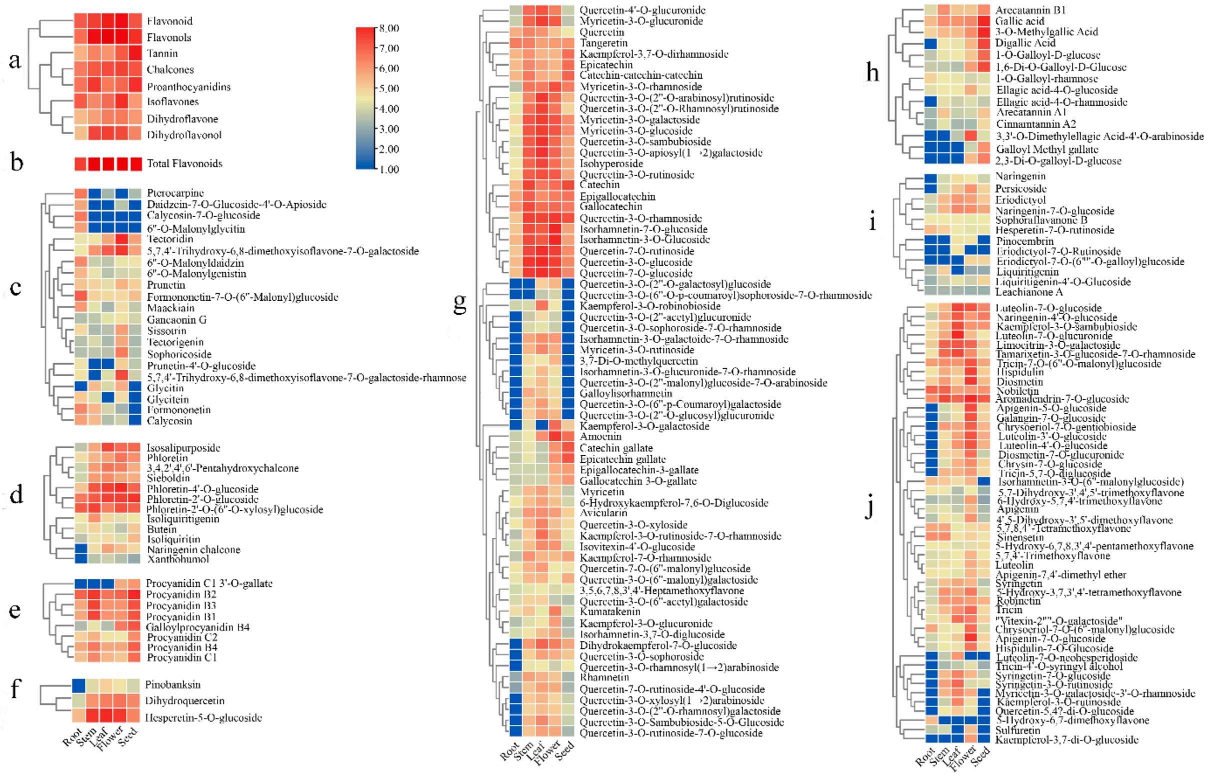

Figure S3. Flavonoids redirected in five different tissues of *C. speciosa* via targeted metabolite analysis. Heatmap of each component contents (a), total flavonoids (b), isoflavones (c), chalcones (d), proanthocyanidins (e), dihydroflavonols (f), flavonols(g), tannins (h), dihydroflavones (i), and flavonoids (j), respectively. The metabolite contents were scaled using Z-score of peak area (mean value of three biological replications) in the heatmap.
